# Nanocarrier-Based Therapeutic Strategies in Myocardial Ischemia–Reperfusion Injury: A Systematic Review of Preclinical Evidence

**DOI:** 10.3390/biomedicines14040921

**Published:** 2026-04-17

**Authors:** Michał Porada, Bartosz Pawełczak, Karolina Barańska-Pawełczak, Krzysztof Marciniec

**Affiliations:** 1 Students’ Scientific Society, Department of Organic Chemistry, Faculty of Pharmaceutical Sciences in Sosnowiec, Medical University of Silesia, Jagiellonska 4, 41-200 Sosnowiec, Poland; 2 Department of Pharmacology, Institute of Medical Sciences, University of Opole, ul. Oleska 48, 45-052 Opole, Poland; 3 Department of Cardiology, Specialistic Hospital in Zabrze, Marii Curie-Skłodowskiej 10, 41-800 Zabrze, Poland; 4 Department of Organic Chemistry, Medical University of Silesia, Jagiellonska 4, 41-200 Sosnowiec, Poland; kmarciniec@sum.edu.pl

**Keywords:** nanocarrier-based drug delivery, myocardial ischemia–reperfusion injury, preclinical animal studies, cardioprotection, oxidative stress

## Abstract

**Background/Objectives**: Myocardial ischemia–reperfusion injury (MIRI) remains an ever-growing threat in the field of cardiology, as it has become a major risk factor for unfavorable outcomes following reperfusion therapies. Oxidative stress and inflammation remain the key pathophysiological mechanisms underlying MIRI, and the presently available treatments fail to prevent this process effectively. This systematic review aimed to summarize and critically assess the latest preclinical research (2020–2026) on nanocarrier-based interventions targeting oxidative stress in MIRI, highlighting the potential of the new nanostructures in cardioprotection. **Methods**: A total of 24 studies meeting the PRISMA criteria have been found through a literature search of PubMed, Embase, and Web of Science databases published between 2020 and 2026. The studies eligible for inclusion had focused on the efficacy of nanocarrier-based interventions in preclinical studies of MIRI. **Results**: Of the 24 included studies, all investigated nanocarrier-based interventions in preclinical models of MIRI. In vitro, ex vivo, and in vivo models were diverse, with most studies being a combination of both in vitro and in vivo models. Commonly studied were lipid-based nanocarriers, polymeric nanoparticles, and biomimetic nanocarriers. Across studies assessed for this review, treatments with nanocarriers were seen to suppress inflammatory and oxidative stress pathways, with a few studies showing a suppression of cardiomyocyte apoptosis. Cardiac function was restored as determined by echocardiography analyses or ex vivo models of the myocardium, thus validating that the nanocarrier-mediated therapies are effective against MIRI. **Conclusions**: The analyzed preclinical studies indicate that the described therapies could provide a promising basis for future clinical trials in the treatment of MIRI, provided their safety and efficacy are confirmed in clinical trials.

## 1. Introduction

Among cardiovascular diseases, ischaemic heart disease remains the most common cause of both morbidity and mortality. The World Health Organization estimates that ischaemic heart disease was the main global cause of mortality, resulting in over 9 million deaths in 2016 [1]. Its most clinically dynamic manifestation, i.e., acute myocardial infarction, requires urgent intervention to prevent prolonged cardiomyocyte hypoxia and, consequently, cardiomyocyte necrosis [2,3,4]. Early successful percutaneous coronary intervention (PCI) significantly reduces short-term mortality in patients with myocardial infarction (MI); however, a substantial proportion subsequently develop heart failure (HF) [5]. While reperfusion of ischaemic tissue is essential for survival, it also initiates secondary damage known as myocardial ischemia–reperfusion injury (MIRI), characterized by excessive inflammation and reactive oxygen species (ROS) in the myocardium, which can subsequently lead to HF [6,7,8,9,10]. Precise global epidemiological data for MIRI are not available, as it is not classified as a distinct clinical entity. However, its clinical relevance is reflected by the occurrence of coronary no-reflow, a manifestation of severe microvascular reperfusion injury [11,12]. No-reflow has been reported in approximately 2–44% of patients with STEMI undergoing primary PCI, depending on the diagnostic criteria and assessment method used [12]. Although no-reflow does not represent the full spectrum of MIRI, its reported incidence provides an indirect estimate of the epidemiological burden of severe reperfusion injury in STEMI patients. The lack of precise epidemiological data also highlights the limited characterization of risk factors predisposing patients to more severe forms of MIRI. One study identified type 2 diabetes mellitus, prolonged pre-hospital delay, and extended door-to-balloon time as factors associated with the occurrence of ischemia–reperfusion injury in patients with STEMI [13]. MIRI primarily involves oxidative damage, cell death, and a profound inflammatory response from the immune system [14,15,16]. Additionally, MIRI is also accompanied by tissue damage, cardiac fibrosis, and cardiomyocyte apoptosis. The ischemia/reperfusion (I/R) injury process inevitably led to massive cardiomyocyte loss, leaving the cells in a state of terminal differentiation and unable to regenerate. Consequently, heart dysfunction and the resulting compensatory fibrosis led to pathological remodeling of the myocardium [17]. In addition, fibrosis plays a key role in determining the prognosis of MIRI-induced heart damage [18]. The underlying mechanisms of MIRI are complex and primarily involve mitochondrial dysfunction, intracellular calcium overload, oxidative stress damage, and the inflammatory cascade [19,20,21].

MIRI in experimental models can account for up to 50% of the final size of the myocardial infarction [22,23]. This illustrates how important it is to provide appropriate therapeutic methods that not only prevent but also modulate this process. In the literature regarding MIRI, therapeutic interventions can be divided into two main categories: pharmacological and non-pharmacological interventions [24]. Non-pharmacological interventions, primarily including ischaemic conditioning, aspiration thrombectomy, left ventricular unloading, and therapeutic hypothermia, have shown limited benefits in clinical studies [24,25,26,27] after initially favorable results in animal studies in cases of hyperoxemia [28] and hypothermia [29]. However, current pharmacological interventions focus on reducing damage and increasing protection against reperfusion injury [24].

Preclinical studies are being conducted on pharmacological interventions that may positively affect MIRI with substances such as Calenduloside E [30], Sappanone A [31], Donepezil [32]—their main mechanism of action is to maintain the dynamic balance of mitochondrial homeostasis, Cyclosporine A [33,34], TRO40303 [35]—their main mechanism of action is to inhibit the opening of mitochondrial permeability transition pore, Kariporide [36], Caldaret [37]—which inhibit calcium overload. In preclinical studies, we can also identify molecules that inhibit inflammation induced by neutrophils, such as CU06-1004 [38], reduce macrophage-mediated inflammation—Iminostilbene [39], Baicalin [40], regulate autophagy—resveratrol [41], cryptotanshinone [42] as well as molecules that improve microvascular surgical damage such as telmisartan [43], Glaucocalyxin A [44], Nicorandil [45] or correct metabolic disorders—CBM-300864 [46], Trimetazidine [47]. We can also distinguish pharmacological intervention strategies in the case of non-classical mechanisms such as long non-coding RNA (lncRNA), which play a significant role in MIRI. It has been shown that lncRNA NONMMUT072211, also known as cardiac ischemia–reperfusion interactive lncRNA ku70 (CIRKIL), interacts with Ku70, both in vivo and in vitro, inhibiting its nuclear translocation [48]. This interaction impairs the repair of double-strand DNA breaks, thereby exacerbating MIRI. Consequently, it has been demonstrated that CIRKIL inhibition alleviates heart injury in vivo, as confirmed by studies conducted on adult ventricular cardiomyocytes and human induced pluripotent stem cell-derived cardiomyocytes [48]. Clinical trials are also being conducted, for example, with Cyclosporine A (NCT01650662) (NCT01502774) or Nicorandil (NCT03445728), but only a few pharmacological therapies have shown promising results in clinical studies. There is generally a lack of clinically effective therapies for the treatment of MIRI.

Strategies to limit the extent of ischemia can be implemented at multiple stages of acute myocardial infarction, including prior to reopening the culprit coronary artery. Evidence suggests that mechanical unloading of the left ventricle (e.g., with an intra-aortic balloon pump, IABP) before reperfusion reduces post-reperfusion edema and the associated activation of signaling cascades that lead to myocardial injury [49]. However, to deliver agents/particles that modulate MIRI to the ischemic territory, vascular patency must first be restored. Immediately upon re-establishing flow in the previously occluded coronary artery, ischemic postconditioning can be applied, consisting of alternating balloon inflations and deflations within the reperfused vessel, which mitigates post-reperfusion edema driven by the surge in blood flow following prior ischemia [50].

It is suspected that oxidative stress, referring to the accumulation of a large amount of ROS, is currently one of the main factors involved in the pathophysiology of MIRI [51,52]. ROS can damage cardiomyocytes in various ways, such as directly attacking cells, causing necrosis, or activating the redox signaling pathway to induce cellular apoptosis [51,52]. ROS, often considered as a toxic product of aerobic metabolism and a major cause of macromolecular damage in MIRI, are rapidly produced during reperfusion [18]. The mitochondrial respiratory chain and NADPH oxidase from the NOX family are the main sources of ROS in cardiomyocytes [18].

The complex and multifaceted pathophysiology of MIRI makes it challenging to develop effective therapeutics. Many pathophysiological mechanisms have been suggested, emphasizing primarily the key role of the endothelium [53]. Despite several extensive experimental and clinical studies on the prevention of MIRI, no specific drug has found routine clinical applications [53]. The main limitations associated with the use of drugs in MIRI concern issues such as drug biodistribution, unfavorable biotransformation, short half-life, and insufficient tissue specificity [54,55]. However, rapid advancements in drug delivery systems, particularly in nanocarrier systems, offer hope for the direct delivery of therapy to the affected regions of the myocardium at the appropriate time and dose [22]. Nanomaterial-based therapies have become a breakthrough strategy for MIRI therapy, demonstrating incomparable benefits in time-dependent drug delivery, multimodal modulation, and bioimaging integration [53].

By exploiting size-dependent engineered biodistribution, enabling endothelial penetration of the myocardium in the 20–50 nm range, combined with stimulus-responsive payload release and biomimetic targeting [53], these nanoplatforms achieve 8–12-fold higher accumulation in the infarcted myocardium compared to free drugs in preclinical models [53]. Careful consideration of nanoparticle properties, including size, shape, and surface chemistry, is essential to minimize off-target accumulation, particularly in the liver and kidneys [56]. Consequently, targeted nanocarrier-based strategies offer a highly promising approach for MIRI therapy, combining enhanced efficacy with low systemic toxicity and improved tissue specificity [22].

Many preclinical investigations have demonstrated the effectiveness of various strategies in mitigating MIRI, but their translation into clinical practice is still limited due to the multifaceted nature of cell death mechanisms and the limitations of single-target therapies [15,16,57,58]. Preclinical studies confirm that exogenous administration of hydrogen sulfide (H_2_S) to damage myocardium can be an effective treatment strategy for MIRI due to H_2_S having many physiological functions, including anti-inflammatory, anti-apoptotic, and mitochondrial protective effects [18,59,60]. The challenge lies in the controlled and prolonged release of H_2_S, which greatly restricts its clinical application [61]. However, many biological responses to H_2_S are dose-dependent, ranging from physiological and protective effects at low concentrations for cells to cytotoxic action at high concentrations [62]. Therefore, controlled-release H_2_S donors are essential to ensure that other tissues are not damaged during the MIRI treatment process [59]. This problem can be solved by using nanocarrier-based drug delivery systems (NDDS), which are a product of applying nanotechnology in medicine. They can increase biomembrane permeability, improve targeted drug delivery, enhance drug stability, prolong the duration of drug action in vivo, and reduce toxicity and improve bioavailability [63,64].

Moreover, other promising gas therapies in MIRI treatment that we should consider include therapies based on nitric oxide (NO) or carbon monoxide (CO). Studies show that NO and its derivatives, such as inhaled NO, nitrites, and peroxynitrite, modulate ischemia–reperfusion injury of the myocardium [65]. Another preclinical study showed that long-term administration of nitrates increased resistance to MIRI in female rats. This was associated with increased eNOS expression in the myocardium and circulating progesterone before ischemia, as well as increased iNOS induced by ischemia and decreased eNOS after MIRI [66].

However, reports on the potential application of CO have been appearing in the literature for many years [67]. Carbon monoxide-releasing molecules (CORMs) are a group of chemical compounds capable of controlled CO release directly into tissues or organs [68]. One study using CORM-3 reported that CO induces delayed cardioprotection against MI, similar to that observed in the late phase of ischemic preconditioning (PC) [69]. Mice were pre-treated with CORM-3 or i-CORM3 (which does not release CO) and subjected to coronary occlusion/reperfusion 24 h later [69]. In mice administered CORM-3, a significant reduction in apoptosis markers (cleaved lamin A, cleaved caspase-3, and cleaved PARP-1) was observed after ischemia–reperfusion injury [70].

**Figure 1:**
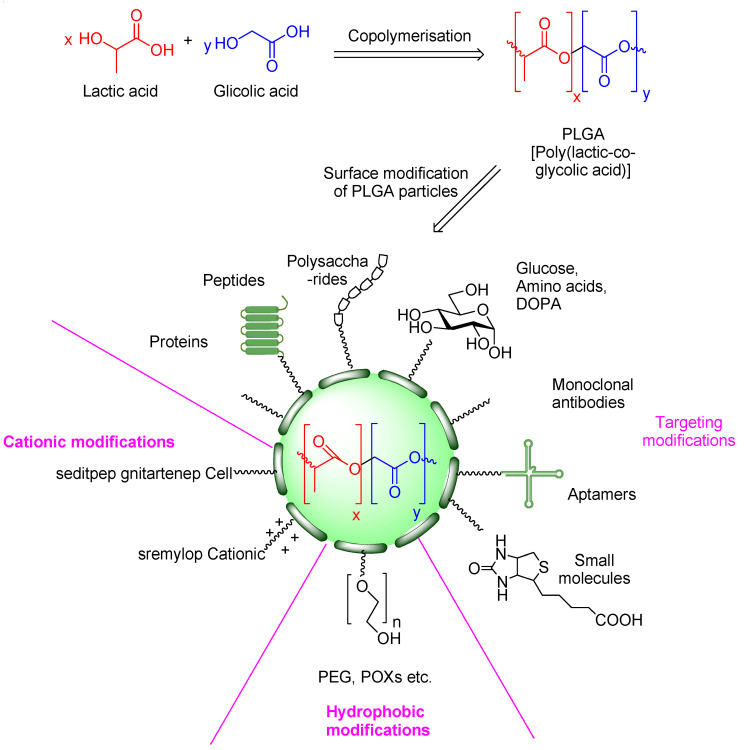
Chemical structure and possible modifications of PLGA [72].

Among them, nanoparticles are widely used. Nanoparticles are spherical formations with an average particle size below 1000 nm [71]. Their nanoscale structure is characterized by the unique exhibiting increased surface area to volume ratios, which enhance their properties as drug carriers. Medicines can be bound to the polymer matrix by dissolving, mechanical entrapping, or encapsulating processes. They include liposomes, niosomes, and quantum dots. PLGA copolymer (Figure 1) is an FDA-approved nanoparticle carrier material that is safe, non-toxic, has good biocompatibility and biodegradability, and its degradation products in the human body can be excreted through metabolism [72,73]. The process of drug release from the polylactide polymer matrix can be easily controlled by the alterations in the PLGA:drug molar ratio [74]. These substances are encapsulated in PLGA nanoparticles or absorbed onto their surface, which greatly prolongs their presence in the body [72,73]. These properties make them promising carriers in MIRI therapy.

Platelet membranes, in combination with PLGA, have recently received considerable attention in the design of target-specific nanoparticles for diagnosing and treating MIRI based on the unique ability of platelets to selectively target and accumulate within injured regions of the myocardium in early stages of MIRI [18]. Platelet activation and accumulation in MIRI are primarily mediated by ligand-receptor interactions, GPIb-IX-V complex engagement with von Willebrand factor released by endothelial cells, αIIbβ3 engagement with αvβ3 subunits of endothelial cells, intracellular adhesion molecule 1 interaction with its respective receptors, P-selectin interaction with endothelial cells’ P-selectin glycoprotein ligand 1 (PSGL-1), and α2β1 or GPVI binding to collagen [75].

Utilizing the selective targeting and immune-modulating properties of platelets, platelet-inspired carriers for diagnosing and therapeutic drug delivery to injured cardiomyocytes have been identified as promising therapeutic modalities for MIRI [76,77,78]. Certain therapeutic agents, such as iron, can have both protective and pathological effects depending upon the particular biological scenario and dose, and can include mechanisms mediated by hydroxyl radical production and ferroptosis [79].

The rationale for this study is based on the rapidly expanding body of preclinical research investigating nanocarrier-based therapeutic strategies for MIRI. Despite this progress, the available evidence remains fragmented with heterogeneous study designs, nanocarrier platforms, and reported outcomes, making it difficult to draw consistent conclusions regarding their efficacy and translational potential. Furthermore, there is currently a lack of an up-to-date systematic review of preclinical data in this field. This review aims to provide a comprehensive overview of preclinical research on nanotherapy for MIRI, with a particular focus on their therapeutic efficacy, cellular and molecular outcomes, safety, and biocompatibility.

## 2. Materials and Methods

A systematic literature review was conducted using the PubMed, Embase, and Web of Science databases to find recent preclinical studies investigating the development of novel and targeted nanoparticle-based drug delivery platforms, utilizing inflammation and oxidative stress modulation. The last search was performed on 4 March 2026 for all databases. No additional databases were consulted. This systematic review was conducted in accordance with the PRISMA 2020 guidelines and was prospectively registered in the PROSPERO database (registration ID number: CRD420251269560).

We searched for articles that had been published within a narrow timeframe between 2020 and 2026, limited to studies published in English. The search strategy utilized the MeSH terms and was conducted according to the PRISMA guidelines. The selection process was also conducted in accordance with PRISMA guidelines and independently by two reviewers who screened all titles, abstracts, and full texts. Any discrepancies were resolved through discussion and consensus to minimize selection bias. No automation tools were used in the selection process. In this review, we included only preclinical studies investigating nanocarrier-based therapeutic interventions in models of MIRI. Specifically, studies conducted in in vitro, ex vivo, and in vivo animal models were considered. Only studies that evaluated therapeutic efficacy, cellular/molecular mechanisms, or safety/biocompatibility of nanocarrier systems were included in this review. The outcomes of interest included therapeutic efficacy (infarct size reduction, improvement in cardiac function), cellular and molecular mechanisms (oxidative stress markers, inflammatory cytokines, signaling pathways), safety, and biocompatibility (cytotoxicity). Only full-text articles published in English were considered with a clear methodology. We excluded review articles, editorials, conference abstracts, as well as studies not related to MIRI or not involving nanotechnology-based interventions.

The search strategy used in this review included a combination of search techniques, using terms that were relevant to (a) nanocarrier platforms, including liposomes, lipid nanoparticles, polymeric nanoparticles and PLGA nanoparticles, (b) targeting approaches, including peptide ligands/RGD sequences or Angiopep-2 and targeting platelet membranes and endothelium, and (c) clinical endpoints, including cardiovascular disorders, inflammation and oxidative stress. To increase relevance to the cardiovascular system, related queries were used. These included words “heart”, “cardiac”, “myocardial infarction”, “ischemia/reperfusion injury”, and “endothelial dysfunction”.

The literature included in this review had to utilize in vitro or in vivo models. Reviews, editorials, and abstracts that did not clearly describe their methodology were excluded, as well as conference reports and literature unrelated to the biology of the cardiovascular system. The data and results were synthesized and presented in a structured table (Table 1). All included studies contributed to a single narrative synthesis, with findings organized descriptively based on predefined study characteristics, including experimental model, nanocarrier type, and outcomes.

Data extraction was performed independently by two reviewers using a standardized approach. Extracted data included study characteristics, nanocarrier type, experimental model, and key outcomes. Any discrepancies were resolved through discussion and consensus. No automation tools were used in the extraction process.

Outcomes are reported using study-specific effect measures, including infarct size expressed as percentage reduction or area at risk, cardiac function parameters, and biochemical markers presented as absolute or relative changes between experimental and control groups.

Methodological quality of preclinical animal studies was rated using SYRCLE’s risk of bias assessment tool for animal intervention studies. The risk of bias was rated under the following domains: sequence generation, baseline characteristics, allocation concealment, random housing, blinding of care providers and outcome assessors, incomplete outcomes data, selective outcomes reporting, and other sources of bias. The domains were rated as having low, high, or unclear risk of bias. The two authors were independent in rating methodological quality, with any disagreements resolved by consensus.

## 3. Results

**Figure 2:**
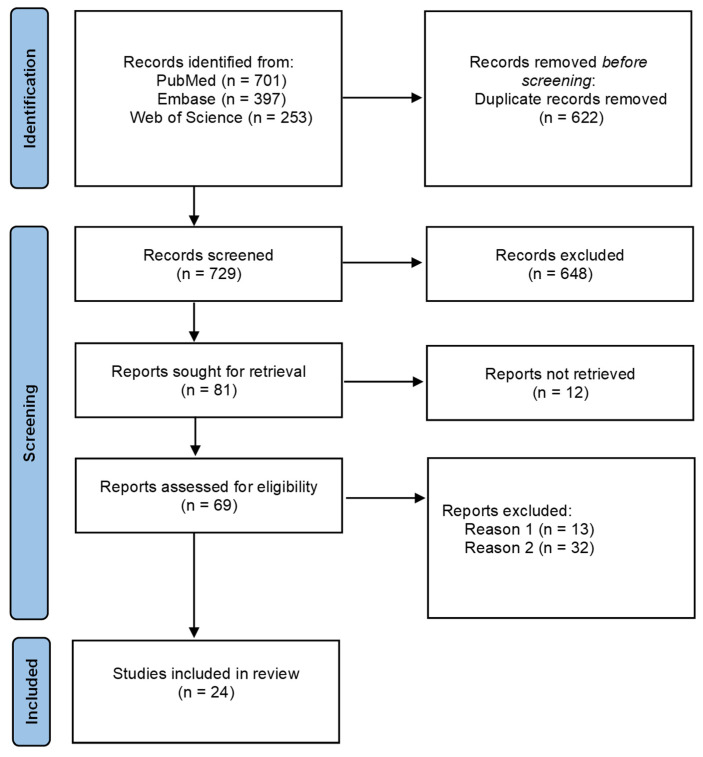
The PRISMA diagram describes the search and selection procedure we used for the overview. (1) Cardiovascular studies not related to MIRI. (2) Non-cardiac studies.

By applying selected queries, we were able to identify 1351 publications, 622 of which were duplicates and were removed. The remaining 729 articles were screened by title and abstract, resulting in the exclusion of 648 papers. A total of 69 publications were assessed in full text for eligibility. Ultimately, 24 studies met the inclusion criteria and were included in our review. The reasons for excluding the remaining 45 studies are presented in the PRISMA diagram (Figure 2).

### 3.1. Characteristics of Included Studies

All 24 publications included in the review concern preclinical studies. The studies were conducted on in vitro, in vivo, and ex vivo models. Most of the work was conducted using two different models by simultaneously studying, for example, an in vitro and an in vivo model. In vivo studies were conducted on Langendorff, C57BL/6, or Sprague-Dawley (SD) mice, or on an ex vivo model using Wistar male rats. MIRI models were obtained by ligation of the left anterior descending (LAD) coronary artery. The included studies investigated a heterogeneous range of nanocarrier platforms, including polymer-based nanoparticles (predominantly PLGA), lipid-based nanostructures such as niosomes, biomimetic platelet membrane-coated systems, and other functionalized nanoparticle formulations. Therapeutic interventions delivered via nanocarriers included hydrogen sulfide donors as well as other bioactive agents targeting inflammation, oxidative stress, apoptosis, and post-ischemic cardiac remodeling.

### 3.2. Therapeutic Strategies and Nanocarrier Platforms

A type of nanoparticle based on polymer represented the most commonly used carrier form in the studies mentioned above, particularly PLGA- and PEG-based systems, which were frequently selected due to their established biocompatibility and flexibility for functional modification. In particular, five of the studies utilized PLGA or PLGA-based formulations due to the well-known biocompatibility and biodegradable characteristics of these materials.

**Figure 3:**
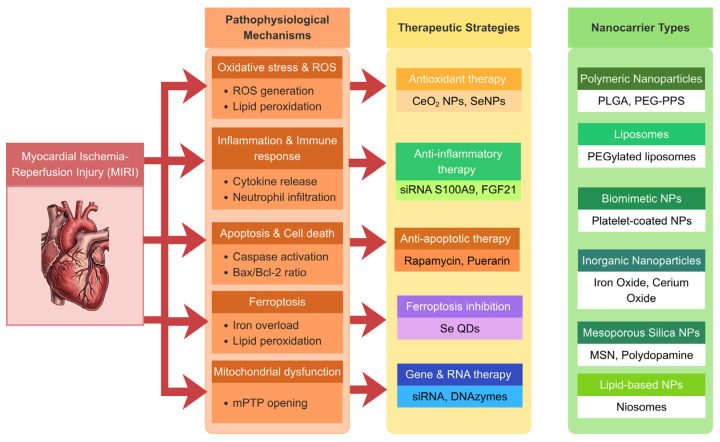
Overview of MIRI pathophysiology, therapeutic modalities, and nanocarrier platforms.

In a considerable number of studies, biomimetic approaches, including macrophage, neutrophil, and platelet membrane-coated nanoparticles, mainly aim at improving targeting of inflamed or ischemic myocardium. Additionally, lipid-based nanocarriers, particularly liposomes, were used, primarily in more advanced and dual-targeting platforms. Lastly, some research used nanoparticles with intrinsic therapeutic properties, such as cerium oxide and selenium-based nanoparticles, which possess nanozyme properties and do not require a drug payload. An overview of the main pathophysiological mechanisms of MIRI and the corresponding therapeutic strategies using nanocarrier systems is presented in Figure 3.

In all of these studies, various nanocarriers were used for targeting a wide range of therapeutic compounds. Nanocarriers were used to deliver a broad range of therapeutic agents, including small molecules, proteins, and siRNA, as well as to exert direct biological effects. While apoptosis, mitochondrial dysfunction, and ferroptosis were also investigated, most of these strategies focused on reducing oxidative stress and inflammation. A comparative summary of targeting strategies and biodistribution profiles across nanocarriers is presented in Table 2.

**Table 1:** Comparison of targeting strategies and biodistribution of nanocarrier platforms in MIRI.

Platform Type	Studies	Targeting Strategy	Evidence of Cardiac Targeting	Biodistribution Profile
Polymeric nanoparticles (PLGA, PEG-based, polymersomes)	[80,81,82,83,84,85]	Passive, peptide-targeted (IMTP) [81], ROS-responsive [81,83], aptamer-conjugated [80]	Moderate, increased infarct accumulation shown for IMTP-modified NPs (NIR imaging) [81], improved myocardial retention reported [85], uptake shown in vitro [80]	Limited, partial data on myocardial retention [85]
Biomimetic nanoparticles (membrane-coated)	[18,59,86,87,88,89,90]	Active targeting via platelet, macrophage, or neutrophil membranes	High, preferential accumulation in ischemic myocardium was demonstrated (organ distribution, IVIS imaging) [59,86,87,88,89]	Moderate, biodistribution assessed (IVIS/organ distribution), but off-target accumulation (liver, kidney) reported [86]
Liposomal systems (including biomimetic liposomes)	[56,91,92]	Dual targeting (mitochondrial TPP, ischemic peptide IMTP) [92], membrane-coated (neutrophil, platelet) [56,91]	High, confirmed myocardial and mitochondrial targeting (cellular uptake, colocalization, IVIS) [56,91,92]	Partial, biodistribution assessed (IVIS), but limited quantitative PK and long-term distribution data
Inorganic/nanozyme-based systems	[90,93,94,95]	Mostly passive, targeted variants (peptide, antibody) [90,94]	Low to moderate, targeting demonstrated in functionalized systems [94], no clear targeting in ex vivo or non-targeted models [93,95]	Very limited, biodistribution largely not assessed or restricted to local delivery models [90,95]
Selenium-based systems	[96,97]	Passive targeting, intrinsic nanoparticle activity	Moderate, mitochondrial localization and functional effects reported [96,97], but no direct targeting strategy	Limited, biodistribution reported in some studies [97], but no comprehensive PK analysis
Other platforms (niosomes, squalene-based, carbohydrate NPs)	[79,98,99]	Passive targeting, local administration (niosomes) [79]	Low to moderate effects observed, but no clear evidence of active cardiac targeting	Minimal, biodistribution not assessed or not reported in most studies

### 3.3. Effects on Myocardial Injury and Cardiac Function

In the preclinical studies we analyzed in this review, those based on nanocarriers were consistently associated with reduced myocardial damage and improved cardiac functional parameters in experimental models of myocardial injury due to ischaemia and reperfusion. The ex vivo Langendorff perfused mouse heart model demonstrates that supplementation with PLGA-encapsulated P7C3 nanoparticle concentrations of 100 nM resulted in a significant reduction in the infarct size when compared to control-buffer-treated groups as revealed by TTC staining [80]. Similar results for the limitation of infarct size have also been obtained when free P7C3 solution at 10 µM concentration was used [80]. In a mouse MIRI model, treatment with hesperadin-loaded poly(serine ester phosphate) nanoparticles conjugated with ischemic myocardium-targeting peptide (HI@PSeP-IMTP) resulted in a substantial improvement in cardiac function, as measured by transthoracic echocardiography. These improvements included an increase in LVEF, LVFS, and a decrease in LVDd, LVDs, and LVESV as compared to those in controls treated with PBS [81]. The efficacy of HI@PSeP-IMTP far exceeded that of hesperadin, as well as poly(serine ester phosphate) nanoparticles conjugated with ischemic myocardium-targeting peptide (PSeP-IMTP), used separately, as evidenced by a substantial reduction in fibrosis in the left ventricular papillary muscles [81].

In another mouse MIRI model, treatment with platelet membrane-coated poly(lactic-co-glycolic acid) nanoparticles co-loaded with rapamycin and JK-1 peptide (RAPA/JK-1-PLGA@PM) showed a significant improvement in cardiac function as measured by an increase in LVEF and LVFS values and normalization of left ventricular size compared with MIRI controls [59]. Histopathological analysis showed decreased fibrosis and more uniform arrangements of cardiomyocytes upon treatment with RAPA/JK-1-PLGA@PM than in other groups treated with free RAPA (Rapamycin) or other formulations [59]. Dihydromyricetin-loaded poly(lactic-co-glycolic acid) nanoparticle (DMY-PLGA NP) greatly increased the viability of cardiomyocytes in oxidative stress induced by H_2_O_2_ [82]. In particular, cell viability was about 70% in the DMY-PLGA NP group, compared with 40% in the model group [82].

Platelet membrane-coated mesoporous silica nanoparticles loaded with diallyl trisulfide nanoparticles (PM-MSN-DATS NPs) brought about dramatic improvements in cardiac function in MIRI models in rats. Compared with RM-MSN-DATS, DATS, and PBS, the first group (PM-MSN-DATS) showed an increase in LVEF and LVFS (80.48 ± 3.42% and 41.26 ± 5.62%, respectively. Myocardial fibrosis was shown by Masson’s Trichrome staining, with the result indicating that myocardial fibrosis was reduced (4.37 ± 0.53%), providing strong evidential support for efficient biological attenuation of ischemia–reperfusion injuries in the myocardium [18]. Squalene-based nanoparticles loaded with adenosine (SQAd NPs) greatly reduced the area at risk (AAR) and the infarct size in MIRI in mice [99]. After three days, the percentage area at risk was 27.4 ± 10% in the SQAd NP group, while in the adenosine treatment group, it was 41.4 ± 6.5% [99].

Kindernay et al. [93] aimed to investigate in their study whether small amounts of iron could modify the heart’s response to I/R in isolated perfused rat hearts and protect them through ischaemic preconditioning (IPC). There was no significant difference detected in the values of the haemodynamic parameters between the two groups upon stabilization and the initial value at the end of the pre-ischemic administration of the nanoparticle [93]. But the +(dP/dt)max and −(dP/dt)max values in the Fe + IPC group after a period of 15 min of iron infusion were significantly lower as compared to the control (without iron: CI/R) and Fe-PC (iron preconditioned) groups (*p* < 0.05) [93]. In yet another study, a therapeutic strategy targeted DNAzymes to achieve cardioprotection. The cardioprotective role of DNAzymes was assessed using a rat model of MI [79]. A single dose (150 µL) of drug preparations was injected into the MI area [79]. Echocardiographic analysis revealed that rats receiving DNAzymes encapsulated in nano-niosomes showed enhanced heart functions compared to the Myocardial ischemia/reperfusion (MI/R) group [79].

**Table 2:** Characteristics of preclinical studies investigating nanocarrier-based strategies in MIRI (2020–2026).

Author and Year	Experimental Model	Nanocarrier Type and Composition	Therapeutic Cargo	Main Endpoints	Overall Risk of Bias (SYRCLE)	Key Limitations
Sutariya et al. (2024) [80]	In vitro—C2C12 mouse skeletal myoblasts Ex vivo—mouse heart in the Langendorff perfusion model (MIRI, infarct size assessment)	PLGA-COOH nanoparticles, aptamer-conjugated PLGA NPs (A01B RNA aptamer), sustained-release polymeric nanocarrier	P7C3 (Nampt activator, NAD+ salvage pathway modulator)	Cell viability, cellular uptake, wound closure assay, NF-κB activity (TNF-α-induced), infarct size (TTC staining)	Unclear	Lack of in vivo administration, no biodistribution or pharmacokinetic analysis, aptamer targets skeletal muscle rather than cardiac-specific markers
Ma et al. (2025) [81]	In vitro—human AC16 cardiomyocytes, OGD/R injury model (oxygen-glucose deprivation/reoxygenation) In vivo—mouse MIRI model	PLGA-Se-Se-PEG-IMTP nanoparticles, Diselenide bonds (responsive to ROS), PEG (prolonged circulation), IMTP (peptide targeting ischaemic myocardium)	Hesperadin (a cardioprotective drug, a Calcium/Calmodulin-Dependent Protein Kinase II Delta inhibitor) and ROS-responsive diselenide bonds	Accumulation of nanoparticles in the MIRI region (NIR), reduction in ROS in cardiomyocytes and heart tissue, mitochondrial protection, and γH2AX reduction (DNA damage), reducing the size of the infarct	Low	Lack of long-term safety and pharmacokinetic data, no comparison with standard-of-care therapies, and absence of a detailed dosing and administration protocol
Liu et al. (2024) [59]	In vitro—H9C2 rat cardiomyocytes subjected to hypoxia/reoxygenation In vivo—MIRI mouse model induced by ligation of the left anterior descending coronary artery in C57BL/6 mice	Biomimetic PLGA nanoparticles coated with platelet membranes (PM), designed for active targeting of ischaemic myocardium and pH-dependent controlled release	Rapamycin (RAPA) as an mTOR pathway inhibitor and JK-1 as a hydrogen sulfide (H_2_S) donor, encapsulated in PLGA nanoparticles and released in a controlled manner in response to the acidic inflammatory microenvironment	In vitro—cardiomyocyte viability (CCK-8), cell apoptosis (flow cytometry), cellular uptake of nanoparticles, release of cardiac damage markers (LDH, CK-MB), inflammatory cytokine profile (TNF-α, IL-1β, IL-6, IL-10) In vivo—left ventricular contractile function (LVEF, LVFS), organ distribution and heart targeting ability, myocardial fibrosis and remodeling (histological analysis)	Unclear	The study is limited to preclinical models, which restricts the direct translation of the results to clinical conditions. In vivo evaluation of H_2_S monotherapy has not been conducted, which makes it difficult to fully separate the synergistic effects of rapamycin and H_2_S, the lack of long-term assessment of safety and toxicity, and the need for further optimization of nanoparticle dose
Du et al. (2024) [82]	In vitro—H9C2 rat cardiomyocytes with H_2_O_2_-induced oxidative stress model In vivo—healthy SD male rats—pharmacokinetic study after oral administration (without the in vivo MIRI model)	PLGA polymer nanoparticles, prepared by the emulsification and solvent evaporative method, without surface modification or targeting elements	Dihydromyricetin (DMY) is a flavonoid with antioxidant and cardioprotective properties	Cardiomyocyte survival (CCK-8), LDH, MDA, and SOD levels as markers of oxidative damage, PGC1α and PPARα expression (Western blot), in vivo pharmacokinetic parameters (AUC, T_1_/_2_, Tmax) after oral administration	Unclear	Lack of an in vivo ischemia–reperfusion model, limitation of cardiac functional studies solely to in vitro conditions, absence of haemodynamic and histological effect assessment, lack of long-term observation, and absence of data regarding the safety of chronic nanoparticle use
Chen et al. (2025) [18]	In vitro—human umbilical vein endothelial cells (HUVEC) stimulated with TNF-α, H9C2 cardiomyocyte line (cytotoxicity assessment) In vivo—healthy SD male rats, a model of MIRI induced by temporary LAD ligation (45 min of ischaemia + reperfusion)	A biomimetic nanocarrier based on platelet membrane-coated mesoporous silica nanoparticles (PM-MSN)	Diallyl trisulfide (DATS) (a sustained-release H_2_S donor)	Targeted accumulation in the myocardium (DiR fluorescence imaging), the level of ROS in heart tissue, heart function: LVEF and LVFS (echocardiography), degree of myocardial fibrosis (Masson trichrome staining), in vitro cytotoxicity (CCK-8), in vivo biocompatibility (hematological studies and histopathology)	Low	There is a lack of data regarding long-term functional effects after 4 weeks
Brusini et al. (2023) [99]	In vitro—cardiomyocyte cell lines (HL-1, H9C2) and human Peripheral Blood Mononuclear Cells (PBMCs) In vivo—a mouse model of myocardial ischemia–reperfusion (LAD ligation, 30 min of ischaemia + reperfusion)	Squalene-based nanoparticles loaded with adenosine (SQAd NPs)	Adenosine (chemically linked to squalene)	Cytotoxicity (in vitro), platelet aggregation, infarct area, and area at risk, cardiomyocyte apoptosis in the myocardium after 3 and 7 days of reperfusion	Low	Lack of statistical significance for infarct area reduction, high interindividual variability, absence of in-depth molecular analyses, use of a bolus instead of a continuous infusion, and limited translational value of the rodent model
Kindernay et al. (2023) [93]	Ex vivo model of an isolated, perfused rat heart (Wistar male rats) using the Langendorff method, prolonged ischaemia and reperfusion, with an IPC protocol	Magnetic iron oxide nanoparticles (Fe^2+^/Fe^3+^)	Iron in the form of magnetic iron oxide nanoparticles	Assessment of heart function (LVDP, +(dP/dt)max, −(dP/dt)max, LVSP, LVEDP, HR, coronary flow), susceptibility to reperfusion arrhythmias, expression of RISK pathway proteins (p-Akt, p-GSK-3β, eNOS), apoptosis markers (caspase 3, procaspase 3, BAX/Bcl-2 ratio), and GPX4 levels as a ferroptosis marker	Low	Known variability of cardiac functional response in the Langendorff model, no additional improvement in heart function above the standard IPC effect, and limitation to short-term exposure to iron nanoparticles
Naseroleslami et al. (2023) [79]	In vivo—rat model, ischemia/reperfusion of the myocardium induced by ligation of the LAD coronary artery for 30 min, followed by reperfusion	Nano-niosomes (vesicular nanocarriers based on non-ionic surfactants), prepared by the film hydration methods, morphological characterization performed using AFM, particle size 70–110 nm after DNAzyme encapsulation	DNAzyme (an enzymatic oligonucleotide with anti-inflammatory and antiapoptotic effects)	Echocardiographic assessment of heart function, expression of apoptosis markers (Bax, Bcl-2, caspase-3), inflammation markers (TNF-α, IL-1β), and activation of the transcription factor NF-κB were evaluated by Western blot and immunohistochemistry	Unclear	Local administration of the preparation, lack of data on long-term cardioprotective effects
Li et al. (2020) [83]	In vitro—H9C2 hypoxia/reoxygenation In vivo—rat MIRI model LAD ligation/reperfusion, SD rats	ROS-responsive polymeric nanoparticles (PEG-b-PPS; poly(ethylene glycol)-block-poly(propylene sulfide))	Ginsenoside Rg3 (hydrophobic natural compound)	Infarct size (TTC), cardiac function (EF), ROS levels, inflammatory markers (IL-6, TNF-α), apoptosis (TUNEL, Bax/Bcl-2), fibrosis (Sirius Red, TGF-β/Smad), oxidative stress proteins (Sirt1, Nrf2, HO-1)	High	Intramyocardial administration, lack of long-term outcomes, and no large animal model
Lu et al. (2024) [86]	In vitro—MNHCs, HUVECs, SMCs, RAW 264.7 In vivo—mouse MIRI LAD ligation/reperfusion	Macrophage membrane-coated polymeric nanoparticles (pABOL) modified with hemagglutinin (HA) and receptor for advanced glycation end products (RAGE)—commodified macrophage membrane-coated siRNA nanoparticles (MMM/RNA NPs)	SiRNA targeting S100A9 (S100A9-siRNA)	Infarct size (TTC), cardiac function (LVEF, LVFS), mortality, inflammatory markers (S100A9, TNF-α, IL-6, IL-1β), fibrosis (Masson), myocardial injury markers (LDH, cTnI, ANP, BNP), biodistribution (IVIS)	Unclear	Lack of large animal models, short follow-up, lack of clinical validation, accumulation in the liver and kidneys
Chen et al. (2025) [97]	In vitro—H9C2, NRVMs, oxidative stress/H_2_O_2_ In vivo—mouse MIRI LAD ligation/reperfusion + MI model 3 weeks	Spherical PEGylated selenium nanoparticles (SeNPs; SDS/PEG-stabilized)	Lack of classical cargo (selenium-based)	Infarct size (TTC), cardiac function (EF), survival, apoptosis (TUNEL, caspases), oxidative stress (ROS, MMP), mitochondrial function (OCR, mtDNA), inflammation (IL-1β, IL-6, TNF-α), macrophage polarization (M1), fibrosis (Masson), biodistribution	Low	Lack of animal models, lack of comparison with standard therapy, and short-term follow-up
Zhu et al. (2025) [94]	In vitro—hypoxia/reoxygenation (H/R)-treated H9C2 cardiomyocytes In vivo—mouse MIRI model	Mesoporous polydopamine nanoparticles (mPDA) loaded with CeO_2_ nanozyme (Ce@mPDA), PEG-modified, functionalized with cardiac homing peptide (CHP) and triphenylphosphine (TPP), loaded with dexrazoxane (DXZ)	Dexrazoxane (DXZ) + CeO_2_ (antioxidant catalytic activity)	ROS levels, ferroptosis markers, apoptosis, inflammation (IL-1β, TNF-α, macrophage polarization), iron levels (non-heme iron), cardiac function (EF), fibrosis (collagen deposition)	Unclear	Short-term assessment of mechanisms (despite 28 days of functional follow-up)
Fu et al. (2025) [98]	In vitro—H9c2 (H/R and ROS model), HUVEC In vivo—rat model of MIRI (SD rats) Transplantation model: heterotopic heart transplant (BN, Lewis rats)	Carbohydrate-derived nanoparticles (C-NPs)	Lack of classic cargo, C-NPs act as nanoantioxidants (intrinsic activity)	ROS (in vitro and in vivo), antioxidant enzymes (SOD, GPX, CAT), markers of myocardial damage (CK-MB, LDH, cTnI), inflammatory cytokines (IL-1β, IL-6, TNF-α), infarct size (TTC), myocardium function (LVEF, LVFS), apoptosis (TUNEL), transplant survival (Kaplan–Meier)	Unclear	Lack of data on biodistribution, no comparison with other nanomaterials, effect in the transplant model limited (ROS ↓ not significant), only pretreatment (no therapy after reperfusion)
Sun et al. (2025) [95]	In vitro—H9c2 oxygen-glucose deprivation (OGD) and HUVEC In vivo—rat MIRI model (LAD ligation and reperfusion)	Ceria nanoparticles (CeO_2_, CNPs), alendronate-mediated surface functionalization	No drug cargo, intrinsic nanozyme activity	Cardiac function (LVEF, LVFS), infarct size (TTC), serum markers (CK, CK-MB, cTn-I, LDH), ROS levels, oxidative stress markers (SOD, MDA), apoptosis (TUNEL, Bax/Bcl-2, caspase-3), mitochondrial dynamics (Drp1, p-Drp 1), histology (HE), fibrosis (Masson, Sirius Red)	Unclear	Local (intramyocardial) supply—limited translatability, no biodistribution outside the myocardium, short observation time of molecular mechanisms
Rao et al. (2025) [91]	In vitro—H9c2, RAW264.7, 293T-KLB cells In vivo—mouse MIRI model (LAD ligation 30 min and reperfusion (72 h))	Liposomal nanoparticles (DPPC, SSPC, DOPC, cholesterol), encapsulating rhFGF21, coated with neutrophil membrane (NM-NPs)	Recombinant human FGF21 (rhFGF21)	Cardiac function (LVEF, LVFS), infarct size (TTC), serum markers (CK-MB, cTnT), ROS (DCFH-DA), apoptosis (TUNEL, Bax/Bcl-2), inflammation (macrophage infiltration (Liposomal nanoparticles (DPPC, SSPC, DOPC, cholesterol), encapsulating rhFGF21, coated with neutrophil membrane (NM-NPs))), mitochondrial function (ultrastructure (TEM)), biodistribution	Low	Short observation period (3 days), lack of long-term heart function data
Jiang et al. (2025) [87]	In vitro—HUVEC, SMCs, neutrophils In vivo—murine model MIRI, male C57BL/6 mice	Engineered neutrophil membrane-coated PLGA nanoparticles (ENM/RNA NPs)	S100A9-targeting siRNA	Biodistribution and targeting (IVIS, CLSM), cardiac function (LVEF, LVFS), infarct size and fibrosis, inflammation (S100A9, TNF-α, IL-1β, IL-6, MPO), neutrophil recruitment, survival, safety (hemolysis, histology)	Low	Short follow-up, limited assessment of long-term immunogenicity
Li et al. (2025) [96]	In vitro—H9c2 H/R and erastin-induced ferroptosis model In vivo—murine MIRI model (LAD ligation 45 min and reperfusion)	Porous silica nanospheres loaded with selenium quantum dots (Se@PSN), PVP-modified mesoporous SiO_2_ core–shell system	Selenium quantum dots (SeQDs)	Ferroptosis markers (GPX4, SLC7A11, lipid peroxidation, Fe^2+^), mitochondrial function (OXPHOS complexes, membrane potential, ROS), oxidative stress (ROS, NRF2, SOD), cardiac injury (infarct size, LDH, CK-MB, cTnI), cardiac function (EF)	Unclear	Local (intramyocardial) route of administration—low clinical translatability
Lei et al. (2024) [88]	In vitro—HUVECs, RAW264.7 In vivo—mice C57BL/6 MIRI model	Biomimetic platelet membrane-coated nanocrystals (BA NC@PM), baicalin nanocrystals (HPMC-based) coated with plated membrane	Baicalin	Cardiac function (EF), infarct size (TTC), angiogenesis (CD31, VEGF expression), inflammation (IL-1β, TNF-α, IL-10), ROS levels, histology (H&E, Masson), biodistribution, safety (ALT, AST)	Unclear	Short-term observation, lack of precise pharmacokinetic data, and molecular mechanisms not fully explained
Ikeda et al. (2021) [84]	In vitro—bone marrow-derived macrophages (BMDMs) In vivo—mouse MIRI model (C57BL/6J, CypD-KO, CCR2-KO, double)	PLGA-based nanoparticles prepared by emulsion solvent diffusion method: CsA-NP (cyclosporine-loaded PLGA nanoparticles), Pitava-NP (pitavastatin-loaded PLGA nanoparticles), FITC-NP (fluorescent PLGA nanoparticles)	Cyclosporine A, pitavastatin, FITC	Infarct size (TTC staining), area at risk (Evans blue), cardiac injury markers (inferred from infarct size reduction), monocyte recruitment (flow cytometry, Ly6Chigh cells), inflammation markers (IL-1β, IL-18), FMT imaging (cell death, protease activity), NLRP3 inflammasome activation (in vitro macrophages)	High	Intravenous nanoparticle delivery has been tested only in controlled preclinical settings, with short follow-up, limited pharmacokinetic, and long-term toxicity assessment
Altshuler et al. (2021) [85]	In vitro—H9c2 cardiomyoblasts subjected to H/R In vivo—rat MIRI model (LAD ligation)	Porous polymersome nanoparticles composed of 75 mol% PEG-PBD (poly(ethylene glycol)-polybutadiene), 25 mol% PEG-PPO (poly(ethylene glycol)-poly(propylene oxide))	Superoxide dismutase (SOD)	Enzymatic activity of SOD (ferricytochrome c assay), ROS levels (H2DCFDA assay), mitochondrial membrane potential (JC-1 assay), cell viability and cytotoxicity (MTT, LDH), myocardial retention of SOD (in vivo fluorescence imaging), infarct size (TTC staining), lipid peroxidation (MDA levels), LV function (EF)	Unclear	Intramyocardial delivery used, short-term ROS quantification limitations in vivo, no long-term toxicity or immunogenicity evaluation of nanoparticles
Xu et al. (2024) [89]	In vitro—H9c2 (H/R), HUVECs, THP-1, BMDMs In vivo—mouse MIRI model	Platelet membrane-derived nanocarrier (PL720) encapsulating L-arginine and FTY720	L-arginine (NO donor precursor) + FTY720 (S1PR1 agonist)	Apoptosis (TUNEL, Bax/Bcl-2, p-AKT), macrophage polarization (M1/M2 markers: iNOS, CD206, STAT3), cardiac function (EF, LV volumes), fibrosis (Masson staining), angiogenesis (CD31), inflammation (IL-1β, TNF-α, TGF-β, IL-10), biodistribution and targeting, safety (ALT, AST, CREA)	Unclear	No long-term data beyond 28 days
Weng et al. (2022) [56]	In vitro—BMDMs, HUVECs, HL-1 cardiomyocytes In vivo—mouse model of MIRI	Platelet membrane-coated, ROS-responsive liposomes (PLP-RvD1) composed of SPC + cholesterol + DSPE-SeSe-PEG2000, hybridized with platelet membrane vesicles (PMVs)	Resolvin D1 (RvD1)	Cardiac function (LVEF, LVEDV, LVESV), infarct size and fibrosis (Masson staining), biodistribution/targeting (IVIS, IF), angiogenesis (CD31, tube formation), safety (cytokines, organ function)	Unclear	Limited pharmacokinetic analysis (beyond biodistribution)
Cheng et al. (2026) [90]	In vitro—F11 cells, Dorsal Root Ganglion (DRG) In vivo—rat model of MIRI with intraspinal injection	Iron oxide nanocubes (Fe_3_O_4_) coated with DSPE-PEG2000 and conjugated with anti-TRPV1 antibody (FeNCs-TRPV1)	Anti-TRPV1 antibody (targeting TRPV1 channels, neuromodulatory approach)	Infarct size (IS/AAR), serum cTnI, ventricular arrhythmia score, norepinephrine levels, prosurvival kinases (Akt, ERK, GSK-3β), apoptosis (TUNEL, Bax/Bcl-2, caspase-3), spinal signaling (Camkk2, AMPK, SP, CGRP)	Low	Invasive intraspinal delivery, preconditioning model, and lack of long-term safety assessment
Wang et al. (2024) [92]	In vitro—H9c2 cardiomyocytes (H/R) In vivo—C57BL/6J mice MIRI model (LAD ligation, 30 min ischemia + 24 h reperfusion)	Dual-targeted liposomes (PUE@T/I-L): soybean lecithin + cholesterol + TPP-PEG-PE (mitochondrial targeting) + IMTP-PEG-PE (ischemic myocardium targeting peptide)	Puerarin (PUE)	Cellular uptake and mitochondrial targeting, mPTP opening, ROS and SOD levels, cell viability and apoptosis, CK-MB, LDH, infarct size (TTC), histology (H&E, TUNEL), mitochondrial morphology (TEM)	Unclear	

Across polymeric and liposomal platforms, treatment consistently translated into meaningful attenuation of myocardial injury and preservation of cardiac function. In vivo ROS-responsive PEG-b-PPS-Rg3 nanoparticles markedly reduced infarct size and improved echocardiographic parameters, including ejection fraction and fractional shortening, while also limiting ventricular dilation and restoring aortic flow dynamics [83]. These functional improvements were accompanied by reductions in circulating cardiac injury biomarkers such as CK-MB and LDH [83]. Similarly, enzyme-loaded polymersomes (NP-SOD) significantly decreased acute ischemic damage and preserved left ventricular function, outperforming both the free enzyme and control groups [85]. Importantly, these benefits extended into the chronic phase, where nanoparticle-treated animals exhibited reduced scar burden and improved hemodynamic performance, including enhanced contractility [85]. Liposomal formulations further reinforced these findings, with mitochondria-targeted systems (PUE@T/I-L) demonstrating the strongest reductions in infarct size and biochemical injury markers, alongside clear improvements in myocardial histology [92]. Additionally, nanoparticle-based dual-targeting strategies (CsA-NP and Pitava-NP) showed additive infarct-sparing effects, particularly when both mitochondrial injury and inflammation were simultaneously addressed [84].

Similar effects were observed on various biomimetic platforms. MMM/RNA/NPs improved LVEF and LVFS, reduced infarct size, fibrosis, and cardiac injury markers, and also lowered mortality [86]. NM-NP rhFGF21 similarly improved functional regeneration, restoring myocardial structure, enhancing LVEF/LVFS, and reducing infarct size and cardiac enzyme levels, as well as improving mitochondrial function [91]. ENM/RNA NPs confirmed these effects, demonstrating sustained improvement in heart function, reduced fibrosis, and improved survival [87]. BA NC@PM also improved heart function and limited the expansion of the infarct, while supporting structural regeneration [88].

Platelet membrane-based systems further strengthened these results. PL720 significantly reduced the size of the infarct and improved LVEF and LVFS, particularly in dual-phase treatment, preserving the ventricular structure [89]. PLP-RvD1 similarly improved LVEF and LVEF, reduced ventricular remodeling and scar formation, and increased angiogenesis [56].

Zhu et al. [94] showed that a hierarchically targeted CeO_2_-loaded mesoporous polydopamine platform lowered oxidative stress, apoptosis, reduced fibrosis, and improved long-term echocardiography parameters, with DXZ-loaded formulation showing the strongest effect. Similarly, Sun et al. [95] reported that ceria nanoparticles decreased infarct size, reduced serum markers of myocardial injury, and improved left ventricular systolic function, with benefits persisting to day 28 and accompanied by less fibrotic remodeling. PEG2000-modified particles performed best in vivo. Cheng et al. [90] further demonstrated that mitochondria-targeted liposomes carrying puerarin reduced CK-MB and LDH release, limited infarct size, improved myocardial histology, and decreased cardiomyocyte apoptosis with dual-modified PUE@T/I-L formulation outperforming free puerarin and non-targeted controls.

SeNPs reduced cardiomyocyte death and limited inflammatory infiltration, with clear mitochondrial localization supporting their direct cytoprotective action [97]. Similarly, Se@PSN significantly improved cardiac function (EF, FS), reduced infarct size, lowered serum injury markers (LDH, CK-MB, cTnI), and attenuated both early injury and late-stage fibrosis [96]. FeNCs-TRPV1, applied as a magnetothermal preconditioning system, markedly reduced infarct size and circulating cTnI levels, while also decreasing the incidence of ventricular arrhythmias [90].

### 3.4. Cellular and Molecular Outcomes

Preclinical models of myocardial injury provide a valuable platform to investigate cellular and molecular mechanisms underlying cardioprotection. P7C3 encapsulated in PLGA nanoparticles showed a significant modulation of release profiles compared to free P7C3, which was released entirely within 24 h, compared to five days for nanoparticulate systems. A01B aptamer-conjugated nanoparticles showed a dose-dependent increase in cell uptake compared to untreated nanoparticles [80].

HI@PSeP-IMTP treatment strongly reduced cardiomyocyte apoptosis both in vitro and in vivo, as well as reduced ROS production induced by OGD/R injury, which is consistent with reduced superoxide anion levels in ischemic myocardium. In addition, this formula also reduced inflammatory infiltration, which was reflected as reduced M1 macrophage number and reduced γH2AX expression, which indicates reduced DNA damage in cardiomyocytes [81].

Similarly, RAPA/JK-1-PLGA@PM reduced apoptosis of cardiomyocytes in H9C2 cells subjected to a hypoxia/reoxygenation environment, together with decreased ROS production and maintenance of mitochondrial function. The formulation also suppressed pro-inflammatory cytokines (TNF-α, IL-1β, IL-6) while increasing anti-inflammatory IL-10 levels in vitro [59].

Consistent with this, DMY-PLGA nanoparticles reduced oxidative stress by reducing LDH and MDA levels, as well as by improving SOD activity, while simultaneously inducing PGC1α and PPARα gene expression, thus contributing to the protection of mitochondria and antioxidant effects. Also, DMY-PLGA was found to be significantly more effective than free DMY [82]. The PM-MSN-DATS nanoparticles diminished oxidative stress in cardiac tissue, whereas targeted delivery increased accumulation in the infarcted myocardium, thus playing a protective role at the cellular and molecular levels [18].

The SQAd nanoparticles significantly suppressed pro-inflammatory cytokines (IL-1β, TNF-α, IL-12, IL-17) and improved the regulation of anti-inflammatory cytokines (IL-2, IL-4, IL-10, G-CSF), with increased expression of iNOS [99].

The mechanistic analysis regarding iron homeostasis showed that there was a reduced level of caspase-3 in Fe-PC and Fe + IPC, without any changes to procaspase-3 levels or BAX/Bcl-2 ratio. The reduced level of GPX4 in Fe-PC might represent an increased process of ferroptosis, where both iron deficiency and excess have been attributed to mitochondrial damage with resultant cardiomyocyte damage [93].

Polymeric and liposomal systems exerted cardioprotection through coordinated modulation of oxidative stress, mitochondrial integrity, and inflammation. A central mechanism was the reduction in ROS, observed across all platforms, including PEG-b-PPS-Rg3, NP-SOD, and mitochondria-targeted liposomes [83,85,92]. Mechanistically, Rg3 released from ROS-responsive nanoparticles directly interacted with FoxO3a, restoring antioxidant signaling pathways (Sirt1/PGC-1α/SOD1) and stabilizing mitochondrial membrane potential [83]. Similarly, NP-SOD preserved mitochondrial polarization and reduced lipid peroxidation, highlighting the importance of sustained antioxidant activity [85]. Targeted liposomes further enhanced intracellular delivery, promoting lysosomal escape and mitochondrial accumulation, which translated into effective inhibition of mPTP opening and downstream ROS generation [92].

PEG-b-PPS-Rg3 suppressed NF- κB activation and reduced systemic and myocardial levels of pro-inflammatory cytokines [83], while nanoparticle-mediated targeting of CCR2-dependent monocyte recruitment significantly attenuated inflammatory cell infiltration [84]. Notably, combined targeting of mitochondrial dysfunction and inflammation produced additive effects, as demonstrated by dual nanoparticle strategies (CsA-NP + Pitava-NP), which reduced both cell death and inflammatory signaling [84]. These interventions also converged on apoptosis-related pathways, decreasing caspase activation, downregulating pro-apoptotic proteins (Bax) and increasing anti-apoptotic mediators (Bcl-2), ultimately limiting cardiomyocyte loss [83,92].

Biomimetic systems exerted strong anti-inflammatory, antioxidative, and cytoprotective effects. MMM/RNA and ENN/RNA NPs suppressed S100A9 and downstream cytokines (TNF-α, IL-6, IL-1β), reducing inflammatory amplification and immune cell recruitment [86,87]. NM-NP rhFGF21 reduced ROS production, inhibited NF- κB signaling, and decreased apoptosis (Bax/Bcl-2) [91]. BA NC@PM reduced pro-inflammatory cytokines, increased IL-10, scavenged ROS, and promoted angiogenesis [88].

PL720 further demonstrated anti-apoptotic activity via AKT activation and enhanced NO production, improving perfusion [89]. Both PL720 and PLP-RvD1 promoted macrophage polarization toward the M2 phenotype (via STAT3), reducing inflammation [56,89]. Additionally, PLP-RvD1 enhanced efferocytosis and increased SPM production, supporting resolution of inflammation and tissue repair [56]. Both systems leveraged monocyte/macrophage interactions to enhance targeting and therapeutic effects [56,89].

At the cellular level, Zhu et al. [94] found that CeO_2_-based nanozymes lowered mitochondrial ROS, reduced apoptosis, and modulated ferroptosis-related signaling, as shown by increased GPX4 and reduced ACSL4 expression; these effects were strongest with myocardial and mitochondrial targeting combined with dexrazoxane loading. Sun et al. [95] similarly showed reduced ROS, increased SOD, decreased MDA, and lower apoptosis, together with upregulation of Bcl-2, downregulation of Bax, and cleaved caspase-3 and preservation of mitochondrial morphology with reduced p-Drp1/Drp1 ratios. Cheng et al. [90] complemented these observations by showing that mitochondria-targeted liposomes inhibited mPTP opening, reduced mitochondrial ROS, increased SOD activity, improved H9c2 viability, and decreased apoptosis while also demonstrating efficient lysosomal escape and mitochondrial colocalization.

SeNPs inhibited the STAT1-ROS amplification loop, reducing the oxidative stress, inflammatory cytokine production, and macrophage M1 polarization, while limiting mitochondrial damage and apoptosis [97]. Se@PSN primarily targeted ferroptosis and mitochondrial dysfunction, restoring the SLC7A11/GPX4 axis, enhancing OXPHOS-related gene expression, reducing lipid peroxidation, and activating PI3K/Akt signaling [96]. These effects were accompanied by improved antioxidant defenses (NRF2, SOD2) and reduced ROS accumulation. In contrast, FeNCs-TRPV1 operated through a neurocardiac axis, where controlled TRPV1 activation induced Ca^2+^ influx and downstream Camkk2/AMPK signaling, ultimately leading to desensitization of spinal TRPV1, reduced neuropeptide release (SP, CGRP), and activation of prosurvival kinases (Akt, ERK, GSK-3β) [90].

Finally, DNAzymes encapsulated in nano-niosomes reduced the expression of pro-apoptotic factors Bax and caspase-3 and pro-inflammatory mediators TNF-α, IL-1β, and NF-κB, while increasing the anti-apoptotic protein Bcl-2 compared with MI/R alone [79].

### 3.5. Safety and Biocompatibility

The safety and biocompatibility of preclinical therapeutic strategies are critical for their further translation into clinical applications and eventual testing in human subjects. PLGA nanoparticles of P7C3 showed an excellent safety profile, with no significant cytotoxicity observed towards proliferating C2C12 myoblast cells at various concentrations and time points [80]. HI@PSeP-IMTP showed the retention of cardiomyocyte viability and had a significantly lower rate of apoptosis compared to the free hesperadin and non-targeted PSeP-IMTP formulation. The efficiency of the target delivery, analyzed through PAT and ICP-MS, showed improved and preferential uptake of HI@PSeP-IMTP within the ischemic left ventricular anterior wall with safe distribution, thereby signifying excellent biocompatibility and targetability [81].

RAPA/JK-1-PLGA@PM had an excellent cell viability profile, with minimal cytotoxicity observed towards H9C2 cells, while also having excellent hemocompatibility, with a haemolysis rate of 1.28 ± 0.03% at 700 µg/mL levels in the red blood cell haemolysis test. In vivo distribution assays clearly showed preferential uptake within the ischemic myocardium, with minimal off-targeting observed within the liver, spleen, kidneys, and lungs, thereby signifying excellent biocompatibility and targetability [59]. DMY-PLGA nanoparticles showed minimal cytotoxicity towards H9C2 cardiomyocytes, while having excellent pharmacokinetics, with an improved AUC level of 2.04-fold and an extended half-life of 1.46-fold, indicating improved stability, excellent biocompatibility, and improved pharmacokinetics, thereby signifying improved long-acting efficacy of the formulation compared to the free form of the active molecule, DMY [82].

PM-MSN-DATS showed minimal cytotoxicity towards H9C2 cardiomyocytes, with an approximately 85% cell viability rate observed at a 100 µM concentration, while also showing excellent biocompatibility, with the absence of obvious indicators of inflammation and toxicity observed through hematological and histopathological assays, thereby confirming the excellent biocompatibility of the platelet membrane-coated mesoporous silica nanoparticle platform [18]. SQAd nanoparticles had minimal cytotoxicity towards HL-1 cells, H9C2, peripheral blood mononuclear cells, and platelet cell assays, while also not exhibiting haemolysis and adverse effects, indicating the excellent cytotoxicity profile of the formulation, lacking adverse effects towards peripheral blood mononuclear cells, platelets, and blood cells, thereby indicating the formulation to be safe and well-adaptable towards the targeted therapy of MIRI [99].

The analyzed studies consistently reported favorable safety and biocompatibility profiles for polymeric and liposomal nanocarriers. In vitro assessments demonstrated minimal cytotoxicity across formulations, including NP-SOD and empty nanoparticles, with preserved cell viability and no significant increase in LDH release [85]. Importantly, nanoparticle encapsulation often improved the safety profile of active compounds. Free Rg3 caused high mortality when administered intramyocardially, whereas its nanoparticle-encapsulated form was well tolerated and therapeutically effective [83]. Liposomal systems also showed low haemolytic activity across a wide concentration range, with encapsulation reducing direct membrane toxicity compared to the free drug [92]. Additionally, these platforms exhibited high physicochemical stability in both storage conditions and serum-containing environments, maintaining size distribution and surface properties over time [85,92]. Enhanced myocardial retention without evidence of acute toxicity further supports their translational potential [85].

MMM/RNA NPs showed minimal cytotoxicity and no hemolysis [86], while NM-NP rhFGF and ENN/RNA NPs exhibited no cytotoxicity, normal organ function, and no histopathological changes [87,91]. BA NC@PM similarly showed low toxicity and preserved organ integrity [88]. PL720 and PLP-RvD1 confirmed these findings, with no significant cytotoxicity, organ toxicity, or coagulation disturbances [56,89]. Importantly, controlled release strategies improved safety by reducing off-target effects and drug-related toxicity (FTY-720 induced bradycardia) [56,89].

Sun et al. [95] provided the clearest evidence of biocompatibility, showing that ceria nanoparticles did not reduce H9c2 or HUVEC viability and did not impair hepatic or renal function in rats at either 24 h or 28 days after administration. Cheng et al. [90] additionally showed that the dual-modified liposomes remained stable during storage and in serum and displayed low haemolytic activity, with liposomal encapsulation reducing the haemolytic tendency observed for free puerarin. Zhu et al. [94] mainly reported good nanosystem stability, including preserved particle size and low polydispersity, but provided less extensive biological safety data.

SeNPs exhibited minimal cytotoxicity in cardiomyocytes and showed a broader biocompatibility window compared to inorganic selenium forms [97]. Se@PSN showed low in vitro toxicity, no adverse effects on cardiac function, and no histopathological or biochemical signs of organ toxicity even at supra-therapeutic doses, with complete clearance within 48 h [96]. Similarly, FeNCs-TRPV1 displayed no cytotoxicity in vitro and no structural abnormalities in major organs in vivo, confirming good tolerability under magnetothermal conditions [90].

Finally, there were no adverse effects observed on administering the iron nanoparticles during ischemic preconditioning, while also not affecting the role of the formulation on the recovery of post-ischemia–reperfusion cardiac function and the heart’s resistance to arrhythmia caused due to reperfusion, thereby clearly confirming the absence of any harmful effects on the preconditioned myocardium and the excellent biocompatibility of the formulation, thus being safe and well-adaptable towards the targeted therapy of MIRI caused due to ischemic preconditioning-induced cardioprotection [93]. Physiochemical characterization revealed that nano-niosomes had a size range of 60–90 nm, and after DNAzyme encapsulation, the size shifted to 70–110 nm, indicating a stable formulation with adequate capacity for DNAzyme delivery [79].

## 4. Discussion

The field of nanochemistry in medical research had a long journey from the first described use of liposomal vaccines in 1974 to modern selenium quantum dots conjugated with silica nanoparticles (2024) or angiotensin 1 (2025) in myocardial ischemia–reperfusion injury therapy [96,100,101]. MIRI has remained a major concern for clinicians due to the induction of cardiomyocyte cell death by apoptosis, necrosis, necroptosis, and pyroptosis, alterations in energy metabolism due to mitochondrial damage in the setting of oxidative stress and calcium overload, increased inflammation, and loss of endothelial cells with associated coronary microvascular dysfunction [102]. In recent years, nanotechnology therapeutic approaches have been explored for the management of MIRI due to the major limitations associated with currently available cardioprotective agents, such as poor bioavailability, rapid systemic clearance, and lack of tissue specificity. This systematic review summarizes current preclinical evidence indicating that nanocarrier-based interventions consistently mitigate myocardial damage, improve heart function, and modulate cellular and molecular pathways involved in MIRI, while maintaining favorable safety and biocompatibility profiles.

### 4.1. Cardioprotective Efficacy and Functional Outcomes

Throughout the included studies, the reduction in infarct size and improved functional outcomes of the myocardium after MIRI were always established in relation to the delivery of therapeutic agents via nanocarriers. The parameters of LVEF, LVFS, and ventricular remodeling have been improved in various in vivo and ex vivo models, indicating that nanoparticle-based formulations can effectively preserve myocardial structure and function under ischemic stress [18,59,80,81,82,85,92,99]. This advantage is most likely driven by improved bioavailability and prolonged myocardial retention, enabling effective cardioprotection even at lower doses [80,85].

However, the magnitude of benefit varied depending on platform design. Conventional polymeric and liposomal systems primarily enhanced pharmacokinetics, whereas targeted and biomimetic nanocarriers achieved superior outcomes through increased accumulation in ischemic myocardium, resulting in greater reductions in fibrosis and improved survival [18,59,81,86,87]. In particular, membrane-coated and platelet-based systems enabled more precise and phase-specific effects, combining early cytoprotection with later immunomodulation and repair [56,89].

Conventional polymeric and liposomal systems primarily rely on passive accumulation, resulting in moderate myocardial uptake. In contrast, actively targeted systems such as peptide-, aptamer- or membrane-functionalized nanoparticles, consistently demonstrate superior cardiac accumulation and retention. Among these, biomimetic platforms (platelet- or immune cell-derived membranes) appear the most efficient, as they exploit endogenous homing mechanisms, enabling preferential localization in ischemic myocardium and improved therapeutic outcomes.

Inorganic and nanozyme-based platforms, as well as selenium-based systems, demonstrated comparable functional improvements despite distinct mechanisms, highlighting that effective cardioprotection can be achieved through different therapeutic axes, including oxidative stress reduction, mitochondrial preservation, or ferroptosis inhibition [94,95,96]. Notably, approaches integrating multiple mechanisms consistently produced the most pronounced effects, including attenuation of adverse remodeling and sustained functional recovery [84,85,94].

These findings indicate that while improved delivery is a key determinant of efficacy, the degree of cardioprotection is primarily driven by the ability of nanocarriers to combine targeted delivery with multi-pathway intervention.

### 4.2. Integration of Cellular and Molecular Mechanisms

At all subgroups, cardioprotection consistently results from simultaneous modulation of interconnected pathways: oxidative stress, mitochondrial dysfunction, inflammation, and cell death rather than single-target effects.

Polymeric and liposomal systems primarily highlight tight coupling between ROS reduction and mitochondrial preservation, which translates into downstream anti-apoptotic effects, while also showing that isolated pathway targeting may be insufficient due to feedback between inflammation and mitochondrial injury [83,84,92].

Biomimetic platforms add a distinct advantage through active targeting and biological integration, combining suppression of inflammatory recruitment with antioxidant and anti-apoptotic effects. More advanced systems further incorporate pro-regenerative and immunomodulatory signaling, achieving broader mechanistic coverage than conventional carriers [56,86,89,91].

Inorganic and nanozyme-based systems act more upstream, directly controlling ROS and stabilizing mitochondrial function, which secondarily limits apoptosis and structural damage. These platforms more explicitly integrate ferroptosis regulation into the therapeutic response [90,94,95].

Finally, Se-based and neuromodulatory systems demonstrate the highest level of integration: Se nanoparticles function as intracellular multi-target modulators, while FeNCs-TRPV1 extends cardioprotection to a system-level (neurogenic) mechanism, despite converging on similar downstream pathways [90,96,97].

Differences between platforms lie not in the pathways targeted, but in the level and mode of their integration, supporting the concept that nanocarriers act as multi-target, network-level therapies in MIRI.

### 4.3. Safety, Biocompatibility, and Translational Relevance

A key aspect that needs to be taken into consideration in the translation of nanotherapeutics to the clinical setting is an acceptable level of safety and biocompatibility. The majority of studies included in this review showed minimal cytotoxicity against cardiomyocytes and other target cells, hemocompatibility, and the absence of significant organ toxicity in vivo [18,59,80,82,99]. More importantly, the targeted delivery approaches showed negligible off-target accumulation within the major organs [59,81]. Pharmacokinetic enhancements, such as extended circulation time and increased AUC, have been seen for various nanocarrier formulations, highlighting their stability and sustained therapeutic exposure with minimal toxicities [82].

Importantly, translational feasibility depends on the specific materials used rather than general nanocarrier design. PLGA-based nanoparticles [80,82,84,87] remain the most clinically viable due to their established safety and scalable GMP production. However, functionalization introduces additional challenges. Aptamer-conjugated systems [80] improve targeting specificity but face limitations related to production cost, nuclease sensitivity, and potential immune activation, necessitating chemical stabilization.

In contrast, ROS-responsive systems incorporating diselenide bonds [81] offer disease-specific drug release but present notable translational barriers. Their redox-sensitive chemistry complicates large-scale manufacturing and stability, while selenium-containing degradation products raise concerns regarding toxicity and narrow therapeutic windows. Similarly, safety and regulatory uncertainties apply to selenium-based nanoparticles and quantum dot systems [96,97].

Biomimetic nanocarriers coated with platelet or immune cell membranes [18,59,89,91] enhance targeting and immune evasion, but are limited by challenges in reproducibility, donor variability, and potential immunogenicity, complicating GMP standardization.

Nucleic acid-based approaches, including siRNA and DNAzymes [79,86,87], show strong therapeutic potential but remain constrained by instability, off-target effects, and innate immune activation, requiring complex chemical modifications.

Inorganic and nanozyme-based systems [90,93,94,95] provide potent antioxidant activity but raise concerns regarding long-term accumulation, biodegradability, and interference with pathophysiological redox processes. Furthermore, iron nanocarriers used for the purposes of ischemic preconditioning did not adversely affect either the cardioprotective properties or the arrhythmogenic potential, thus emphasizing based on dosages administration context [93].

Notably, the pathophysiology of MIRI in humans, which may be complicated by comorbidities such as diabetes, aging, and chronic inflammation, varies significantly from what one may adopt in controlled research models.

### 4.4. Limitations of Current Evidence

Despite the encouraging results, there are some drawbacks that must not be overlooked. There is considerable variability with respect to the type of nanoparticles, target approaches, and models used in the studies considered, which do not allow for easy intercomparison. Furthermore, most studies are focused on the short-term results, with little information regarding the functional recovery and chronic toxicity, which have not yet been adequately assessed. In addition, invasive routes (intraspinal or intramyocardial administration) and the use of healthy young animals limit the translational relevance of the findings. The use of small animal models again raises questions regarding the generalizability of findings to human models. Moreover, being largely confined to short-term endpoints, these experiments are liable to yield exaggerated results in terms of therapeutic effectiveness.

In addition to the limitations of the included studies, this review has several methodological limitations that should be considered. The literature search was limited to three databases, which may have led to the absence of significant studies. Moreover, due to experimental designs, animal models, and outcome measures, a formal meta-analysis could not be conducted, which limits the ability to quantitatively synthesize the results.

### 4.5. Future Directions

Importantly, based on current knowledge regarding MIRI and the therapeutic application of nanocarriers, they emphasize the urgent need for a therapeutic strategy that addresses the multifactorial mechanisms underlying MIRI, a gap that nanocarrier systems could potentially fill, thus aiding in therapy. Future research should focus on standardizing experimental approaches to ischemia–reperfusion models and defining endpoints. A direct comparison of nanocarriers from different platforms will help define the optimal nanocarrier platform suitable for delivery to and functioning within the myocardium. Further information will be obtained from long-term safety and repeated-dose studies. Ultimately, combining nanotherapeutic approaches with current reperfusion methods and large animal studies will play an integral role in the successful translation of human trials. In summary, encapsulation of drugs into nanoparticles, such as niosomes formed from PLGA or other polymers, may bring clinically important improvement in MIRI therapy.

## 5. Conclusions

This review summarizes the current preclinical evidence supporting nanocarrier-based therapies as a promising strategy for preventing or mitigating myocardial injury caused by ischemia and reperfusion. Biodegradable polymer-based nanoparticle systems can enhance tissue specificity, prolong drug action, and enable simultaneous modulation of multiple pathological pathways. Experimental studies report improved heart function, preserved cellular homeostasis, and acceptable safety and biocompatibility profiles. However, further standardization and rigorous translational studies are necessary to confirm their clinical application.

## Data Availability

No new data were created or analyzed in this study.

## Abbreviations

The following abbreviations are used in this manuscript:

AMI	Acute myocardial infarction
AUC	Area under the curve
CI/R	Control ischemia/reperfusion
DATS	Diallyl trisulfide
DMY	Dihydromyricetin
DMY-PLGA NP	Dihydromyricetin-loaded poly(lactic-co-glycolic acid) nanoparticle
DOPA	L-3,4-dihydroxyphenylalanine
FDA	Food and Drug Administration
GMP	Good Manufacturing Practice
HF	Heart failure
HI@PSeP-IMTP	Hesperadin-loaded poly(serine ester phosphate) nanoparticles conjugated with ischemic myocardium-targeting peptide
HUVEC	Human umbilical vein endothelial cells
ICP-MS	Inductively coupled plasma mass spectrometry
IMTP	Ischemic myocardium-targeting peptide
IPC	Ischemic preconditioning
I/R	Ischemia/reperfusion
LAD	Left anterior descending
LVDd	Left ventricular end-diastolic diameter
LVDs	Left ventricular end-systolic diameter
LVEF	Left ventricular ejection fraction
LVESV	Left ventricular end-systolic volume
LVFS	Left ventricular fractional shortening
MI	Myocardial infarction
MI/R	Myocardial ischemia/reperfusion
MIRI	Myocardial ischemia–reperfusion injury
MNHCs	Mononuclear hematopoietic cells
NADPH	Nicotinamide adenine dinucleotide phosphate
NDDS	Nanocarrier-based drug delivery systems
NF-κB	Nuclear factor kappa-light-chain-enhancer of activated B cells
NIR	Near-infrared
NRVMs	Neonatal rat ventricular myocytes
NOX	NADPH oxidase
NPs	Nanoparticles
OGD/R	Oxygen–glucose deprivation/reoxygenation
P7C3	1-(3,6-dibromo-9*H*-carbazol-9-yl)-3-(phenylamino)propan-2-ol
PBS	Phosphate-buffered saline
PEG POXs	Polyethylene glycol Poly(2-oxazoline)s
PGC1α	Peroxisome proliferator-activated receptor gamma coactivator 1 alpha
PLGA	Poly(lactic-co-glycolic acid)
PM-MSN-DATS NP	Platelet membrane-coated mesoporous silica nanoparticles loaded with diallyl trisulfide
PPARα	Peroxisome proliferator-activated receptor alpha
PSGL-1	P-selectin glycoprotein ligand 1
PSeP-IMTP	Poly(serine ester phosphate) nanoparticles conjugated with ischemic myocardium-targeting peptide
RAW 264.7	Murine macrophage cell line RAW 264.7
RAPA	Rapamycin
RAPA/JK-1-PLGA@PM	Platelet membrane-coated poly(lactic-co-glycolic acid) nanoparticles co-loaded with rapamycin and JK-1 peptide
RGD	Arginine–glycine–aspartic acid
RM-MSN-DATS NP	Red blood cell membrane-coated mesoporous silica nanoparticle loaded with diallyl trisulfide
ROS	Reactive oxygen species
SD	Sprague-Dawley
SOD	Superoxide dismutase
SQAd NP	Squalene-based nanoparticles loaded with adenosine
SMCs	Smooth muscle cells
(dP/dt)	Rate of change in left ventricular pressure over time
